# Molecular docking analysis of phytoconstituent from Momordica charantia with Guanylate Cyclase catalytic domain

**DOI:** 10.6026/97320630014378

**Published:** 2018-07-31

**Authors:** Mohankrishna Ghanta, Elango Panchanathan, Bhaskar Venkata Kameswara Subrahmanya Lakkakula, Anbumani Narayanaswamy, P.A. Abhinand, Stalin Antony

**Affiliations:** 1 Department of Pharmacology, Sri Ramachandra Medical College and Research Institute- Deemed to be University, Chennai-600116, Tamil Nadu, India; 2 Department of Molecular Genetics, Research Division, Sickle Cell Institute Chhattisgarh, Raipur- 492001, Chhattisgarh, India; 3 Department of Microbiology, Sri Ramachandra Medical College and Research Institute- Deemed to be University, Chennai-600116, Tamil Nadu, India; 4 Department of Bioinformatics, Sri Ramachandra Medical College and Research Institute- Deemed to be University, Chennai-600116, Tamil Nadu, India; 5 Centre for Advanced Studies in Botany and Centre for Herbal Sciences, University of Madras, Guindy Campus, Chennai-600 025, Tamil Nadu, India

**Keywords:** In silico screening, ODQ, soluble Guanylate cyclase

## Abstract

Soluble guanylate cyclase (sGC) is a type of lyase enzyme with profoundly increasing importance in treatments of cardiovascular and neurodegenerative disorders. Modulation of sGC activity demonstrated beneficial effects against Parkinson's disease by reducing glutamate excitotoxicity. It is of interest to evaluate the pharmacological activity of Momordica charantia phytoconstituent (DGalacturonic acid) and ODQ with catalytic domain of sGC enzyme, using Autodock version 4.2 programs. Docking results revealed the binding ability of ODQ at the allosteric sites of sGC. D-galacturonic acid also shows binding interaction at the same allosteric sites in the catalytic domain of sGC like ODQ. Results show that both the ligands have efficient binding to THR 474 amino acid residue of beta 1 subunit of the enzyme. The drug likeliness score further implies the suitability of D-Galacturonic acid as a drug-like molecule. The binding property of ODQ and D-Galacturonic acid with the catalytic domain help to inhibit sGC activity having pharmacological effects. Moreover, ODQ interaction with heme site of sGC is already known while its interaction with the catalytic domain is shown in this report.

## Background

**Figure 1:**
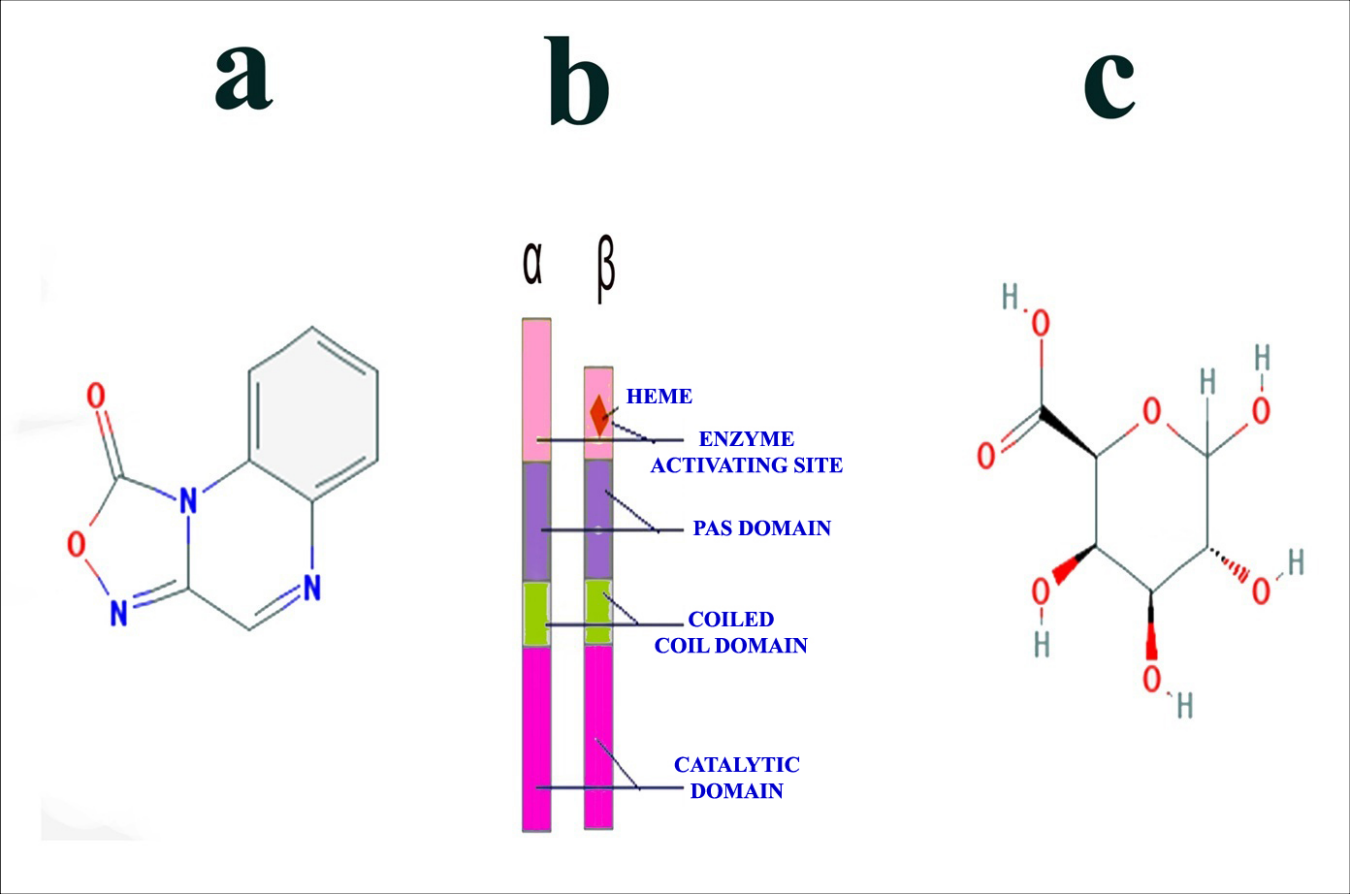
(a) Chemical structure of ODQ, (b) Structure of Soluble Guanylate Cyclase and (c) Structure of D-Galacturonic Acid.

Soluble guanylate cyclase (sGC, EC 4.6.1.2) is a lyase enzyme involved in the synthesis of cyclic guanosine monophosphate (GMP) from guanosine triphosphate (GTP) [1, 2]. It is a hemecontaining heterodimer with alpha and beta subunits [3]. Beta sub unit consist of enzyme activating heme site. But both subunits are required for the enzyme to be activated [4]. The subunits exists in various isoforms namely alpha1, alpha2, beta1, beta2 [5]. The most common subunits in human brain are alpha1 and beta1 [6]. The alpha1 and beta1 subunits of rat sGC contain 690 and 619 amino acids respectively. Truncation studies revealed the architectural information about sGC. The N terminus region of sGC of the β1 subunit is harboring animal heme-binding domain (1-194 residues) and C-terminal region was shown to have catalytic activity (alpha1 467-690 residues and beta1 414-619 residues). Activation of sGC involves binding of the compounds like nitric oxide (NO), carbon monoxide (CO) and oxygen to heme site of beta1 subunit. Catalytic domain of this enzyme is important for binding of important components required for enzyme function. MG+2, a catalyst for this enzyme reaction binds to this catalytic region of sGC [7]. The sGC also contain structures for homologous Per- Arnt-Sim (PAS) and coiled-coil domains in both alpha1 and beta1 subunits (194-385 residues). Although the precise role of these domains is not known, recent research demonstrated that the PAS domain was essential for protein-protein interactions and coiled coil domain was essential for formation of suitable heterodimer [8]. GTP, essential for synthesis of cGMP was reported to bind at catalytic domain [9]. Figure 1B illustrates the allosteric binding sites of the catalytic domain of sGC enzyme [10]. Inhibition of sGC causes reduction in glutamate toxicity and also inhibits apoptosis induced by over regulation of sGC [11, 12].

Momordica charantia Linn. (Cucurbitaceae) is a common medicinal and vegetable plant found in India. The fruits of this plant are used as nutritional and medical supplement. The extracts of Momordica charantia possessed several medical properties such as anti-neoplastic [13], anti-Helmintic [14], anti-Genotoxic [15], antiviral [16], anti-fertility [17], anti-microbial [18], anti-tumorous [16], anti-diabetic [19], and antioxidant activities [20].While a previous study using aqueous extracts of Momordica charantia demonstrated the sGC inhibitory property, did not reveal the exact compound or isolated it. However, the authors stated that the compound was not a lipid [21]. Momordica charantia extract that was processed through the water extraction and alcohol precipitation method showed neuroprotective property. The major component of this extract was D-Galacturonic acid [22]. In our study, a possibility for modulation of the enzyme activity through catalytic domain with D-Galacturonic acid and 1H-[1, 2, 4]oxadiazolo[4,3-a]quinoxalin-1-one (ODQ) is shown using Auto dock tool. D-Galacturonic acid is an uronic acid. Oxidation of the carbon C-6 of D-Galactose forms the corresponding uronic acid called D-galacturonic acid. D-Galactose is a D-aldohexose sugar. D-Galacturonic acid is very important component for many metabolic pathways [23]. The end product of D-galacturonic acid in animals was reported to be 4-deoxy-alpha-L-threo-hex-4-enopyranosyluronic acid constituents [24]. D- Galacturonic acid is one of the constituent of many fruit and vegetable pectin [25]. So it is a very important uronic acid available as water-soluble polysaccharide in the daily foods. Its concentration in various commercial fruit juices is as follows: apple- 43.9 mg/L, mango- 49.4 mg/L, grape fruit- 22.8 mg/L, pear- 16.1 mg/L, clementine- 18.9 mg/L, peach- 23.4 mg/L [26]. ODQ is a selective inhibitor of sGC. ODQ was taken as a standard reference compound to study inhibitory sites of sGC and compared with D-Galacturonic acid binding sites of this enzyme. This inhibitor (ODQ) showed antiparkinsonian activity in preclinical studies [27].

### Chemistry of the Ligands

D-Galacturonic acid, IUPAC name of this compound is (2S, 3R, 4S, 5R)-3, 4, 5, 6-tetrahydroxyoxane-2-carboxylic acid, or Dgalacto- hexopyranuronic acid. Its molecular formula is C6H10O7 [28]. It consists of aldehyde group and carboxylic acid group at C1, C6 positions respectively. The structure of this compound is depicted in Figure 1A.

ODQ, IUPAC name of this compound is [1, 2, 4] oxadiazolo [4, 3- a] quinoxalin-1-one. Its molecular formula is C9H5N3O2 [29]. It is a heterocyclic organic compound with fused structure of a benzopyrazine and oxadiazolone rings. Quinoxaline or benzopyarazine alone contains a ring complex made up of a benzene ring and a pyrazine ring. It is isomeric with other naphthyridines including quinazoline, phthalazine and cinnoline. Quinoxaline derivatives are used as dyes in pharmaceuticals and antibiotics such as olaquindox, carbadox, echinomycin, levomycin and actinoleutin [30]. The other fused ring, oxadiazoles are a class of heterocyclic aromatic chemical compound of the azole family with the molecular formula C2H2N2O. The structure of this compound is depicted in Figure 1C.

## Methodology

### Ligand Preparation and Target Protein Selection

The selected compounds for docking analysis were ODQ and DGalacturonic acid. Pub chem database (http://www.ncbi.nlm.nih.gov/pccompound) was used for obtaining the chemical structures of the compounds. Chem Draw Ultra, 11.0 was used for generating the three dimensional structure of ODQ and D-Galacturonic acid. PRODRG server was used for confirming the structure and minimizing the energy of the ligand molecules [31]. The chemical properties of the ligand were predicted using DataWarrior Software (v04.06.00). Protein Data Bank [PDB], (http://www.pdb.org) was used for retrieving the crystallographic three-dimensional structure of human sGC [PDB ID: 3UVJ].

### Binding site prediction

CastP server was used in this study for searching the possible binding sites of target receptors and predicting the ligandbinding site. Frequently binding and active sites of sGC were coupled with structural cavities and pockets. This is to identify, to obtain measurements of suitable and accessible surface pockets as well as inaccessible cavities, which are deep in terms of area and volume, both in solvent accessible surface and molecular surface. Among the predicted binding site of ODQ and DGalacturonic acid, the small and accurate binding site was selected for molecular docking analysis.

### Docking Analysis

Auto Dock Tools (ADT) version 1.5.6 and Autodock version 4.2 programs [32] (Autodock, Autogrid, Autotors, Copyright-1991- 2000) from (http://www.scripps.edu/mb/olson/doc/autodock) Scripps Research Institute were used to perform this docking analysis. The searching grid was extended above preferred active sites of target proteins. Polar hydrogen charges of Gasteiger-type and Kollman charges were assigned and atomic solvation parameters were added. The non-polar hydrogen was merged with carbon and ligand (ODQ and D-Galacturonic acid) moieties were added with polar hydrogen. Internal degrees of freedom and torsions were set. The compound D-Galacturonic acid and ODQ were docked with target protein complex (PDB ID: 3UVJ) with the ligands being flexible and the protein molecule considered as a rigid body. Computation with default grid spacing of 0.375Å was set for affinity and electrostatic mapping of all atom types present in the protein molecules. Lamarckian Genetic Algorithm was used for docking conformational search and populations of 150 individuals with a mutation rate of 0.02 were evolved for 10 generations. A cluster analysis based on root mean square deviation (RMSD) values, with reference to the starting geometry, was subsequently performed and the lowest energy conformation of the more populated cluster was considered as the most trustable solution. Based on the predicted binding energy, different ligand-protein complexes were sorted and evaluated. PyMol molecular viewer (The PyMOL Molecular Graphics System, Version 1.5.0.4 Schrodinger, LLC) was used for analysing the ligand-protein interactions of selected compounds. Pose View (http://proteinsplus.zbh.uni-hamburg.de/) was used for developing hydrophobic effect of ligands.

## Results

**Figure 2:**
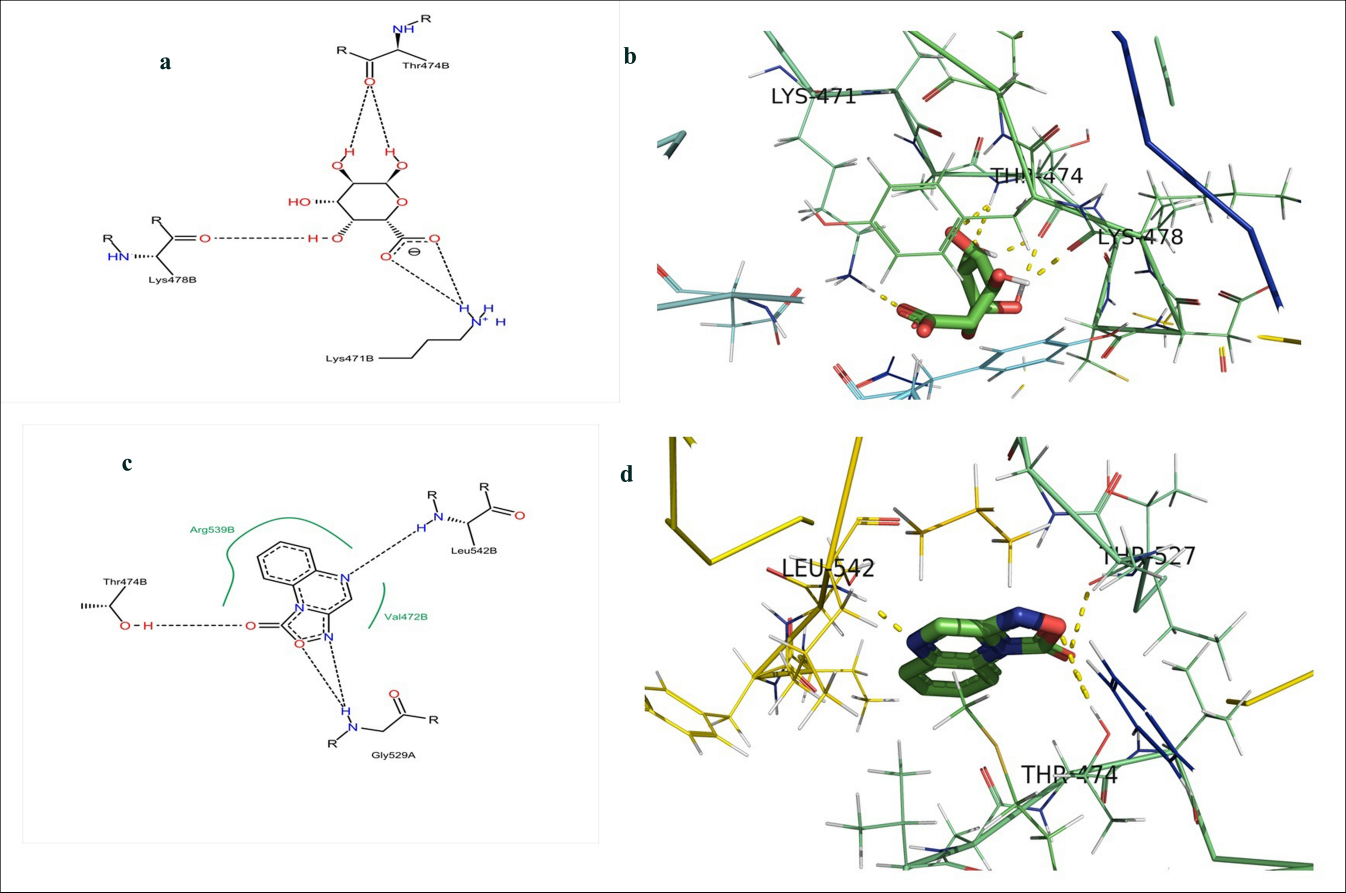
(a) Binding of DGalacturonic Acid with amino acid residues of Soluble Guanylate Cyclase, two-dimensional view. (b) Binding of D-Galacturonic Acid with amino acid residues of Soluble Guanylate Cyclase, three-dimensional view. (c) Binding of ODQ with amino acid residues of Soluble Guanylate Cyclase, two-dimensional view. (d) Binding of ODQ with amino acid residues of Soluble Guanylate Cyclase, three-dimensional view.

D-Galacturonic acid interacted with LYS'471, THR'474, and LYS'478 amino acid residues of beta1 subunit of sGC enzyme (Figure 2A & 2B). ODQ interacted with THR'474, THR'527, and LEU'542 amino acid residues, the first and third amino acid residues are of beta1 sub unit of sGC and second amino acid residue is of alpha subunit (Figure 2C & 2D). THR'474 was the common binding amino acid residue for the both molecules. The binding energies revealed strong binding ability of ODQ to the catalytic amino acid residues of the enzyme. However DGalacturonic acid also showed good binding score to the catalytic binding sites.

### Ligand Efficiency

Ligand efficiency signifies the optimal ligand binding to protein. This depends on the binding energy of the ligand and it is directly proportional. These both ligand interactions were analysed for catalytic domain only. The Ligand efficiency scores are obtained for the ligand interactions. Ligand efficiency measures quantify the molecular properties, particularly size and lipophilicity, of small molecules that are required to gain binding affinity to a drug target. Based on the molecule's polar and nonpolar phase, the log P and log S values were predicted. The values for D-Galacturonic acid were -2.9704, 0.269 and ODQ was 1.507, -2.975. Both ODQ and D-Galacturonic acid did not show any toxicity ratio and the drug likeness score also showed significant values (Table 1). Ligand efficiency of 0.3 was stated as sufficient for a compound to be drug like [33]. In this study ODQ showed a ligand efficiency of 0.45-atom type and D-Galacturonic acid showed a ligand efficiency of 0.35-atom type. Comparatively ODQ has higher ligand efficiency and, both ODQ and DGalacturonic acid ligand efficiency values indicate optimal ligand binding capacity (Table 2).

### Van der Waals Interaction Energy

This Van der Waals contribution to D-Galacturonic acid interactions was higher compared to ODQ, D-Galacturonic acid interactions with protein has sufficient contribution of Van der Waals forces (Table 2).

### Inhibition constant

Inhibition constant values represent dissociation reaction; hence its values are positive. The inhibition constant values were 45.16μM for D-Galacturonic acid and 23.89μM for ODQ. These scores of inhibition constant indicate inhibition of enzyme by both the ligands (Table 2).

## Discussion

The heme part of the beta1 subunit is active site of human sGC. There have been several studies conducted in an attempt to identify the allosteric binding sites of the enzyme that regulate the activity of the sGC. The search for allosteric binding sites is restricted to the catalytic domain of the enzyme. There are several reasons for studying the catalytic domain. It was generally acknowledged that this catalytic domain consisted binding sites for Mg+2 and GTP, and it is also required for the effective dimerization of the enzyme. Any defect or inhibition in this region may lead to the dysfunction of the enzyme. ODQ, a specific Inhibitor that targeted the catalytic domain of sGC, was shown to have antiparkinsonian effect [27]. The unripe fruit extract of Momordica charantia processed through water extraction alcohol precipitation method showed complete inhibition of sGC [21]. Subsequent studies also demonstrated the inhibition of sGC by Momordica charantia extract [34, 35]. D-Galacturonic acid was the major component of Momordica charantia extracts that showed neuroprotection [22]. D-Galacturonic acid known as an uronic acid, very little information is available regarding its biochemical and physiological aspects in human body. Studies revealed the presence of uridine diphosphateglucuronosyltransferases that aid in transport of uronic acid compounds across the blood brain barrier [36, 37]. The in-silico inhibitory activity of D-Galacturonic acid that was demonstrated in the present study seems to support the sGC inhibition by Momordica charantia extractas described in previous studies. Catalytic domain region of beta1 subunit containing threonine amino acid residue at 474 codon forms the hydrogen bonds interfacially to maintain the activity of the enzyme. Disruption of forming these hydrogen bonds may impair the activity of the sGC [38]. The results of this docking study show effective binding of D-Galacturonic acid and ODQ to beta1 THR474 residues, which may impair the activity of the enzyme (Figure 2B). Further, binding of small molecules to allosteric sites of alpha and beta subunits altered its function irrespective of the conformational changes [39, 40]. In-silico screening identified several small molecules that show sGC inhibition by binding to several sites along the dimer interface including the backside pocket of catalytic domain [41]. For two of the molecules that yielded promising docking scores, the ligand-protein interactions were predicted to be with the peptide backbone. These molecules formed hydrogen bonds with the side chain and / or main chain atoms of residues in beta1 S541, L542, T474, and alpha1 G529. They also formed van der Waals interactions with alpha1 Y510, I528, Y532 and beta1 I533, F543 [41]. In the present study, both DGalacturonic acid and ODQ showed significant sGC inhibition scores by binding with beta1 residue THR474. The log P, log S and drug likeness scores also support to this study.

## Conclusion

Docking analysis of D-Galacturonic acid and ODQ showed sufficient binding affinity with sGC catalytic domain and share a common binding site with amino acid residue THR'474. ODQ was known to inhibit sGC through its interactions with heme site of sGC. Results revealed the catalytic domain-binding site of ODQ for regulating the enzyme. It also showed the comparable effects for D-Galacturonic acid as an inhibitor of the sGC with weak binding. This study provides more support for the hypothesis that Momordica charantia extract may have sGC inhibitor activity. Although the D-Galacturonic acid is the principal constituent of Momordica charantia water extract, that exact molecule with more potent sGC inhibition needs to be characterized.

## Conflict of Interest

The authors of this paper have no conflicts of interest, including specific financial interests, relationships, and/or affiliations relevant to the subject matter or materials included.

## Figures and Tables

**Table 1:** Pharmacological properties of Drug compounds.

S.No	Compound properties	D-Galacturonic Acid	ODQ
1	Mol. Weight	194.138	187.158
2	cLogP	-2.9704	1.507
3	cLogS	0.269	-2.975
4	H- acceptor	7	5
5	H-donor	5	0
6	Drug likeliness	-1.2853	-3.2972
7	Mutagenic	None	None
8	Tumorigenic	None	None
9	Reproduction effect	None	None
10	Irritant	None	None
11	Drug Score	0.5991	0.4749

**Table 2:** Factor Scores and Protein-Ligand Complex Formation.

Ligand	Protein PDB ID	Binding amino acid Residues	Binding Energy (kcal/mol)	Inhibition Constant uM	VDW_HB desolv_energy (kcal/mol)	Ligand Efficiency
D-Galacturonic Acid	3UVJ	LYS'471/HZ1 (B), THR'474/HN/O (B), LYS'478/O (B)	-4.55	45.16	-4.78	0.35
ODQ	3UVJ	THR'474/HG1 (B), THR'527/O (A), LEU'542/HN (B)	-6.31	23.89	-6.21	0.45
